# FBPP: software to design PCR primers and probes for nucleic acid base detection of foodborne pathogens

**DOI:** 10.1038/s41598-024-51372-5

**Published:** 2024-01-12

**Authors:** Mohamed A. Soliman, Mohamed S. Azab, Hala A. Hussein, Mohamed M. Roushdy, Mohamed N. Abu el-naga

**Affiliations:** 1 Department of Botany and Microbiology, Faculty of Science (Boys), Al-Azhar University, Nasr City, Cairo Egypt; 2 Department of Radiation Microbiology, National Centre for Radiation Research and Technology, Atomic Energy Authority, Nasr City, Cairo Egypt

**Keywords:** Computational biology and bioinformatics, Microbiology, Molecular biology

## Abstract

Foodborne pathogens can be found in various foods, and it is important to detect foodborne pathogens to provide a safe food supply and to prevent foodborne diseases. The nucleic acid base detection method is one of the most rapid and widely used methods in the detection of foodborne pathogens; it depends on hybridizing the target nucleic acid sequence to a synthetic oligonucleotide (probes or primers) that is complementary to the target sequence. Designing primers and probes for this method is a preliminary and critical step. However, new bioinformatics tools are needed to automate, specific and improve the design sets to be used in the nucleic acid‒base method. Thus, we developed foodborne pathogen primer probe design (FBPP), an open-source, user-friendly graphical interface Python-based application supported by the SQL database for foodborne pathogen virulence factors, for (i) designing primers/probes for detection purposes, (ii) PCR and gel electrophoresis photo simulation, and (iii) checking the specificity of primers/probes.

## Introduction

Foodborne diseases are considered an important cause of mortality and morbidity and a significant obstruction in socioeconomic development worldwide^1^. One of the strategies used to lower the costs and incidence of foodborne diseases is the detection of foodborne pathogens to serve a safe supply of food and inhibit foodborne diseases. The nucleic acid base detection method is one of the most rapid and widely used methods in the detection of foodborne pathogens; it depends on hybridization between the target sequence of the nucleic acid and oligonucleotide primers or probes complementary to the specific nucleic acid sequence of the targeted bacteria^2^.

The design of primers and probes is essential, especially for nucleic acid base detection methods, and it is a preliminary and critical step. This task can be challenging and requires the identification of highly unique conserved regions of target sequences and the ability to display high sensitivity, i.e., the ability to amplify its intended target, and specificity, i.e., the ability not to amplify any nontarget. In addition, the biological parameters of the primers, such as GC content, melting temperature, and the formation of secondary structures, which include self-dimers, hairpins and cross dimers, are essential for evaluating the efficient amplification of a target sequence.

There are a number of software tools available to the public to design primers^3^. However, there are no specific tools for designing primer–probe sets automated for the nucleic acid detection base method. Additionally, these software programs often have limitations in their proficiencies to perform target analysis. Users are typically required to use additional tools for testing primer specificity. They are usually not specific to detect target sequences that contain a significant number of mismatches and are consequently not suitable for application in nucleic acid detection methods. There are several simulation software programs that determine the amplification of amplicons of primer pair targets supplied by users but do not design primers^4^, and some of them are difficult to use without bioinformatics knowledge^5^.

To overcome these issues, we present FBPP, an open-source Python-based application supported with the SQL database for foodborne pathogen virulence factors with the ability to (i) design primers/probes using the modified Primer3 module to be suitable for detection purposes, (ii) perform PCR and gel electrophoresis photo simulations, and (iii) check the specificity of primers/probes. We believe that this application will be particularly useful in nucleic acid base detection methods.

## Method, algorithm and implementation

The FBPP program consists of four modules. The first module is sqlite3^6^, which is used to create and access a database for virulence foodborne pathogen genes, while the Primer3 module^7^, with some little bite modification to be suitable for detection purposes, is used to generate the candidate primer pairs for a given template sequence. Another module for PCR and gel electrophoresis photo simulation is created by using the Pydna module^8^, and the last module for specificity checking uses the BLAST python module Bio. Blast from the Biopython project^9^ to look for matches between the primers and targets and not match with any nontarget region.

Designing primers and probes in FBPP is accomplished in four stages: first, the target sequences are identified; in most situations, they are selected from the program database or through the input section. Next, the modified Primer3 module generates many candidate primer pairs according to the desired primer properties, which are identified by the user or using default primer properties. Then, the candidate primer pairs were subjected to PCR and gel electrophoresis simulation to calculate all amplicons from large lists of primers and ensure that the bands were not false negative results for the primers. Finally, the specificity checking process of success primers in the previous step, by default, uses the BLAST module with specific parameters that ensure high sensitivity such that it can detect a target that contains up to 35% mismatches to the primer sequence to exclude nonspecific primers to avoid false positives. The entire search process can be very long if each pair is searched with BLAST individually. To solve this problem, we saved the BLAST result in the entry database for each primer to avoid repeating the process in every run to the same target sequences, and in case unsaved primer goes to online BLAST, also to avoid being un updated program, all BLAST result for all primer will clear every three months to research again to online update BLAST.

FBPP is implemented in Python and runs on an IDLE 3.7.4 platform. It accesses an SQL database over SQLite 3.10.1. The program was tested on a Windows 10 Home Server with an Intel (R) Core (TM) Processor i5-4210 U with 2.40 GHz and 4 GB RAM.moreover, an EXE file is available so that the user can run the application without the need for a python interpreter, python packages, modules, or any related program such as SQLite.

## Result

### User interface

**Figure 1:**
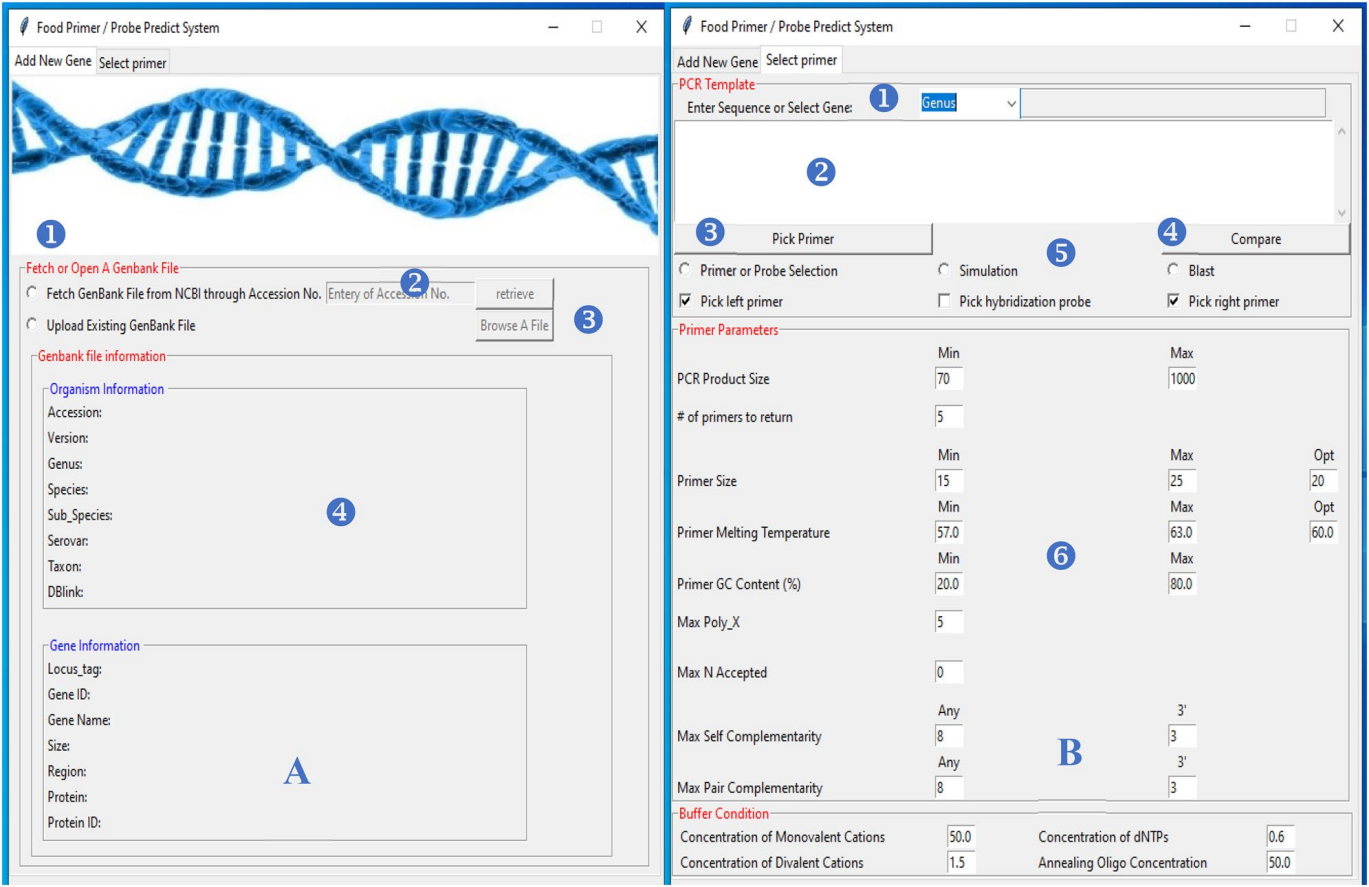
(**A**): Screenshot of “Add New Gene Tab” in the FBPP program: (1) Switch from fetch to upload gene bank file, (2) entry of the accession numbers, (3) browse for the input gene bank file, and (4) the output information of file. (**B**): Screenshot of “Select Primer Tab” in the FBPP program. (1) Select virulence gene from Database, (2) entry of the gene Sequence, (3) click to design the primers or probe, (4) click to compare between template or gene with other sequence in NCBI database, (5) switch between the run options, (6) contains parameters specific to the selected primers and their PCR products or probe, such as the Tm of the PCR product, the primer length, the primer GC content, GC clamps at the 3’-end of the primer, and the PCR buffer conditions.

The interface consists of two tabs; the first tab is “Add New Gene”, where users can insert new virulence genes (Fig. 1-A), and the second tab, “select primer”, has several sections where users can input the gene template, option process and other user-adjustable parameters (Fig. 1-B). In the first tab, there are two ways to enter the virulence gene information into the SQLite database: either by browsing and uploading the GenBank file from the computer or fetching it from NCBI using the accession number.

In the second tab, the user has two options to predict the primer or probe: either by selecting the virulence gene from the program or by entering the sequence of the desired gene. When selecting the gene from the program, the gene information appears on a separate screen. Next, identify the desired properties of individual primers and pairs of primers. These properties include, for example, amplicon (PCR product) length, primer size, melting temperature and GC content. In addition to other options, users can also constrain the complementarity properties of primers to ensure that chosen primer candidates do not bind to themselves and that, in selected primer pairs, the forward primer does not bind to the reverse primer. For convenience, all default choices of parameters have been pretested and are likely to work well in detection applications.

Users have three options: generate the candidate primer pairs for a given template, generate the candidate primer pairs with PCR & gel electrophorese simulation or the specificity checking module uses BLAST in case the template is used from the program. Other options include the compare button to compare the template or gene with other sequences in the NCBI database. can be found under the “PCR Template” section.

### Presentation of results

**Figure 2:**
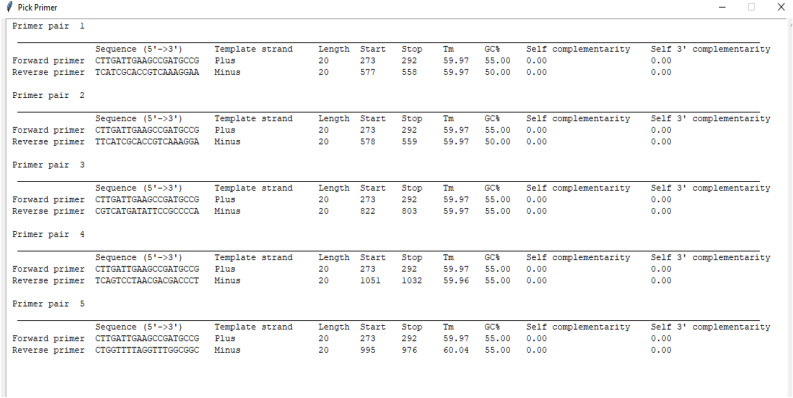
Example results of designing target-specific primers for the invA gene (virulence gene for salmonella), which contains a summary of basic properties for returned primer pairs.

**Figure 3:**
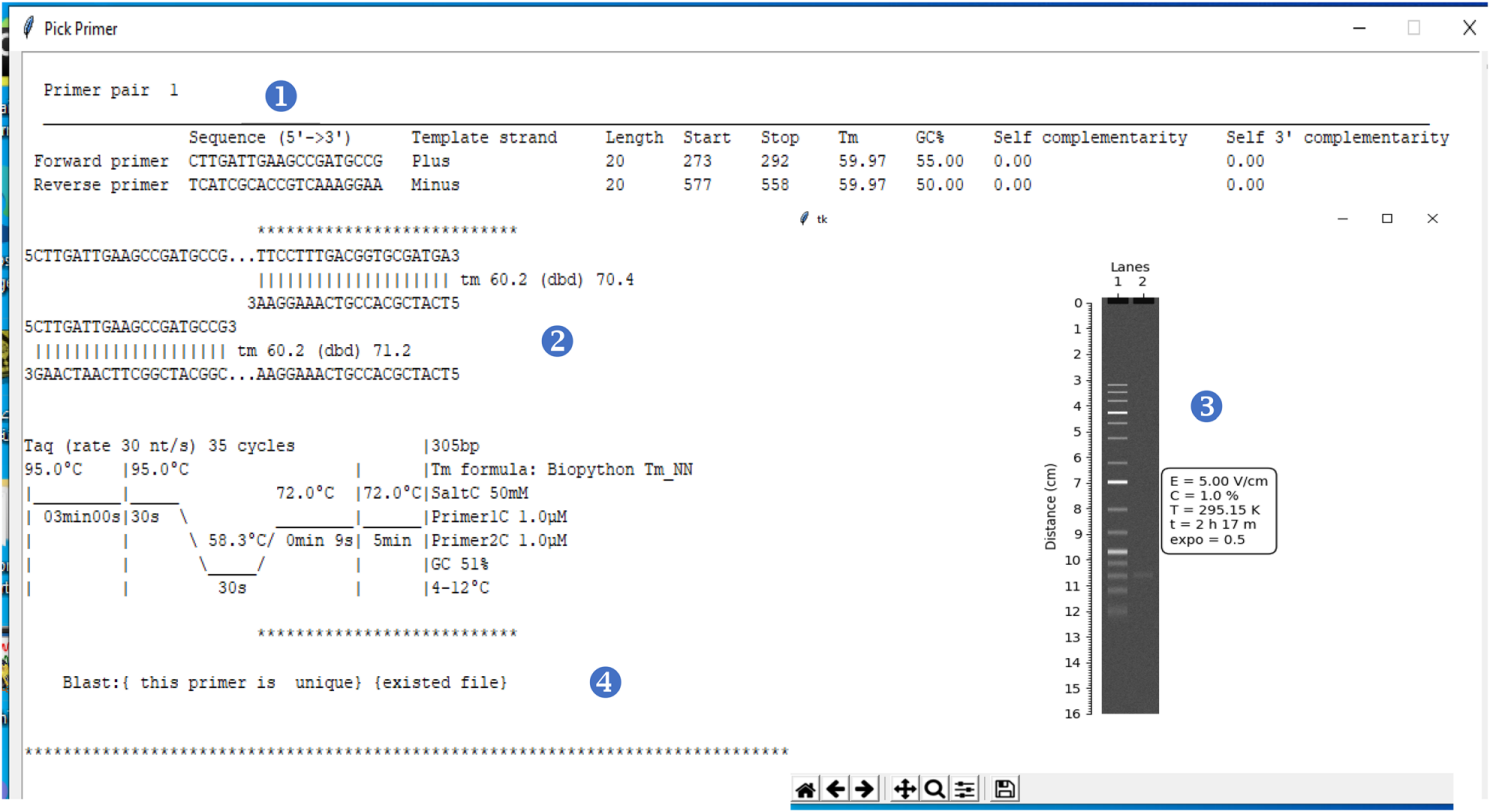
Example results of option Blast for invA gene (virulence gene for salmonella), which contains (1) a summary of basic properties for returned primer pairs, (2) simulation of PCR process , (3) figure of the simulated band, (4) statement of specificity.

According to the user choice, three result reports were obtained. The report of the first option presents the specificity of the generated primers, including sequence, length, position on the consensus sequence for each oligo, Tm, GC content, self-complementarity, and self-3' complementarity (Fig. 2). The report of the second choice (Fig. 3) shows the same information of the first report plus the simulation of PCR process and figure of the observed band. The result of the third option reports the additional statement of specificity checking (Fig. 3).

FBPP offers several features that are not found in other software tools. It is the only tool that specifies the prediction of primer/probe for foodborne pathogenic nucleic acid detection-based methods. In addition, it contains a database for most foodborne pathogen virulence genes; however, the program shows result simulation to avoid false negative results in detection tools.

Another advantage of the FPBP program is the ability to check the specificity of the prime/probe and the number of mismatches that a specific primer pair must have to unintended targets and a custom 3’ end region where a certain number of mismatches must be present.

## Discussion

There are several tools available for the design of primers and probes; however, some of them have limitations that can be mentioned. One of these limitations is that some tools, such as Primer3 (Rozen and Skaletsky, 2000), cannot check specificity and make simulations, which can be accomplished by our program. Although it is a powerful and online tool for designing primers based on a single, short and conserved sequence, the same limitation was found with other software, such as BatchPrimer3^10^, Primaclade^11^, PrimerIdent^12^, GeneFisher^13^, Gemi^5^ and PRISE2^14^.

Primer-BLAST^4^ is one of most famous primer design web browsers provided by NCBI. It combines Primer3 and BLAST along with the Needleman–Wunsch (NW) global alignment algorithm, which overcomes the short ages of local alignment for primer design purposes. Primer BLAST can generate candidate primer pairs and check specificity. However, Primer-BLAST inherits the limitations of Primer3. It does not specify for detection application use, and it has not contained silico PCR, GEL simulation and not define Database for foodborne pathogen which can achieved by FPBP.

Other existing program packages for primer design, including Quant Prime, PRIMEGENS, PRIMEGENS, FastPCR and PrimedRPA, are specific in different applications, such as microarray analysis, DNA methylation pattern analysis of CpG islands, primer location determination, orientation, binding efficiency and primer melting temperature calculation for standard and degenerate oligonucleotides and recombinase polymerase amplification^15–19^.

In this paper, we presented FBPP as a specific-purpose target-specific PCR primer/probe design tool that offers a group of features not found in other tools. It offers primer/probe design, PCR/gel electrophoresis simulation and checks of the specificity of the primer/probe. user-friendly graphical interface, virulence gene database included and pretested default choices for all parameters are provided to be easily usable without skill in bioinformatics or nonmolecular knowledge biologist users, while those more molecular biologists or bioinformatics can use advanced options where these parameters can be highly customized and input gene sequences.

Nucleic acid‒based detection methods are gradually replacing or complementing traditional detection methods in routine microbiology laboratories. We expect FBPP to be a valuable assay design tool for application in these methods, working with highly variable sequences and DNA quality control via PCR.

## Conclusions

In this paper, we presented FBPP as a specific-purpose target-specific PCR primer/probe design tool that offers a group of features not found in other tools. It offers primer/probe design, PCR/gel electrophoresis simulation and checks of the specificity of the primer/probe. user-friendly graphical interface, virulence gene database included and pretested default choices for all parameters are provided to be easily usable without skill in bioinformatics or nonmolecular knowledge biologist users, while those more molecular biologists or bioinformatics can use advanced options where these parameters can be highly customized and input gene sequences.

Nucleic acid‒based detection methods are gradually replacing or complementing traditional detection methods in routine microbiology laboratories. We expect FBPP to be a valuable assay design tool for application in these methods, working with highly variable sequences and DNA quality control via PCR.

## Data Availability

The FBPP Design Tools Desktop application is available here: https://github.com/mohamedmoez1983/FBPP.
